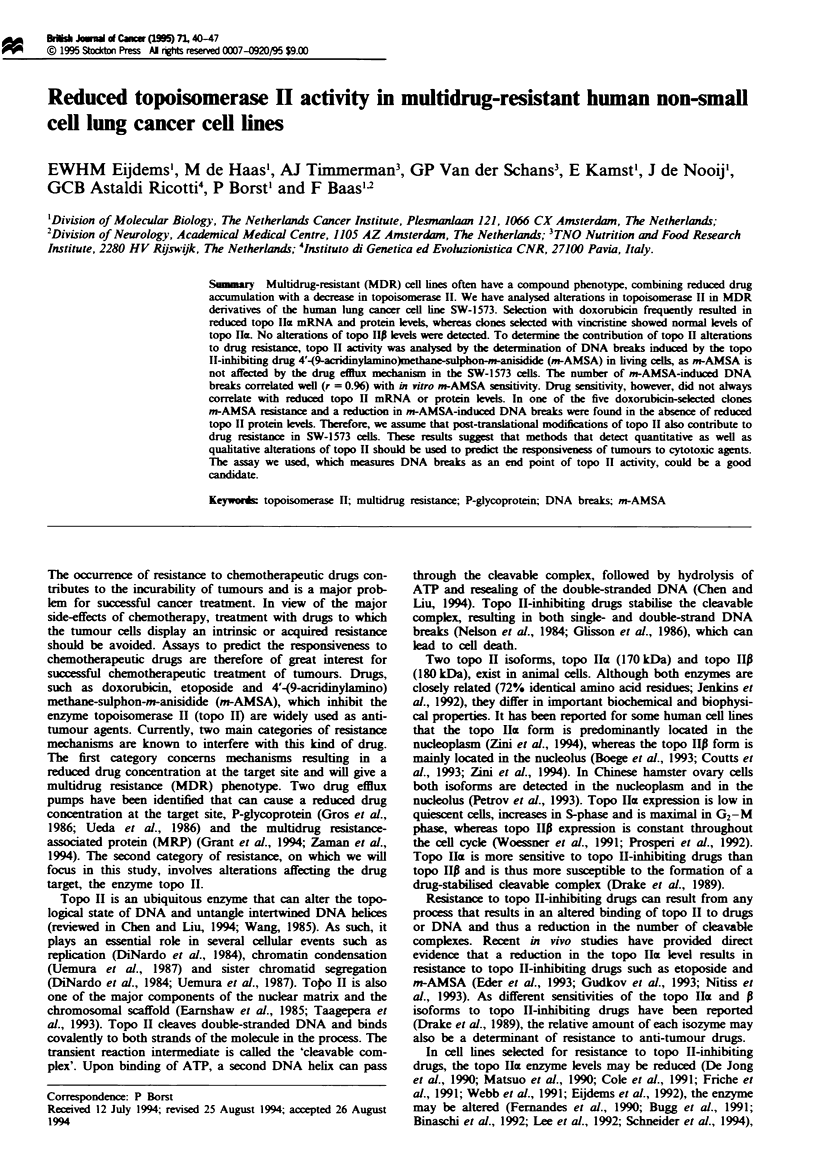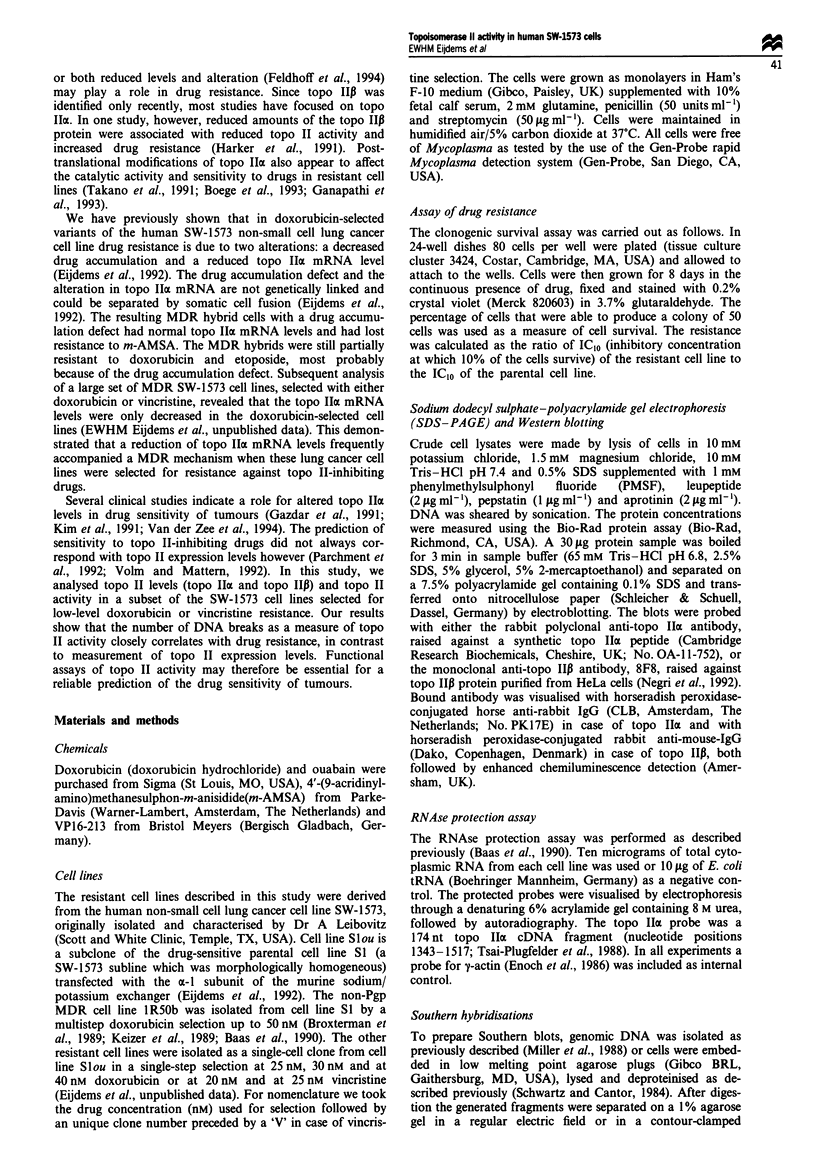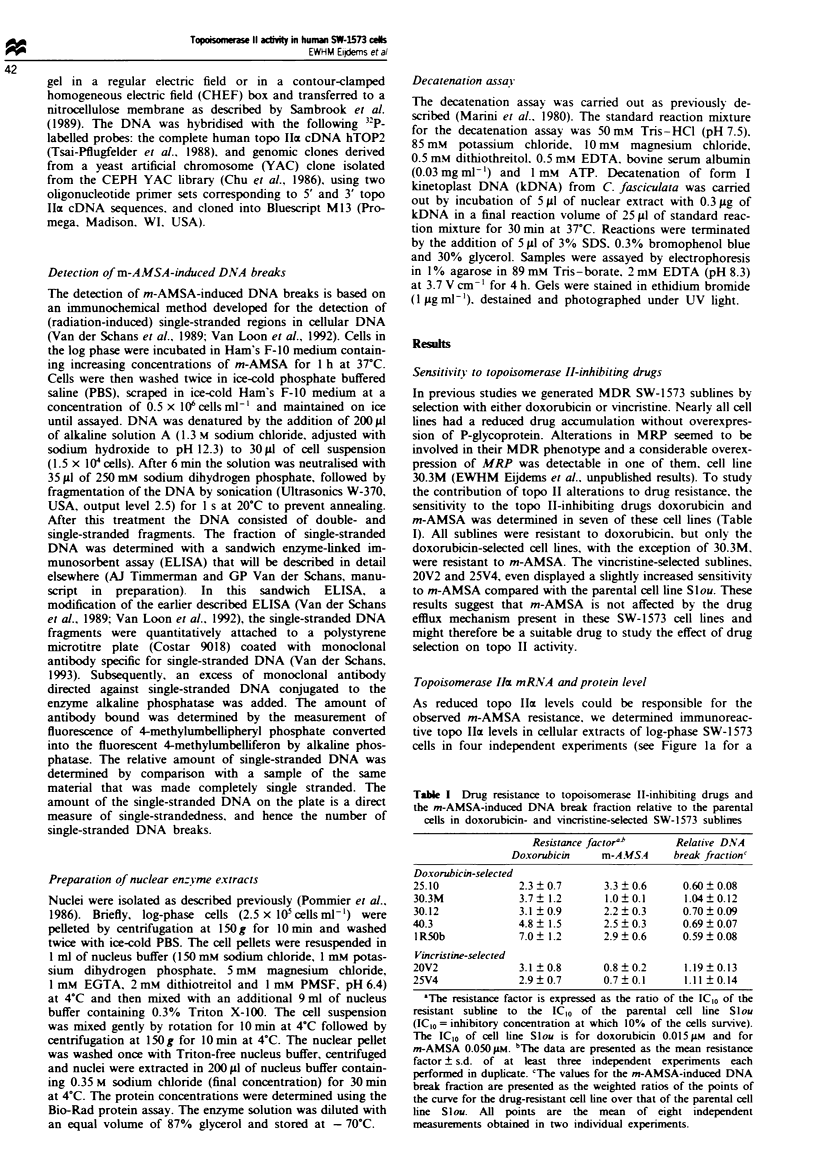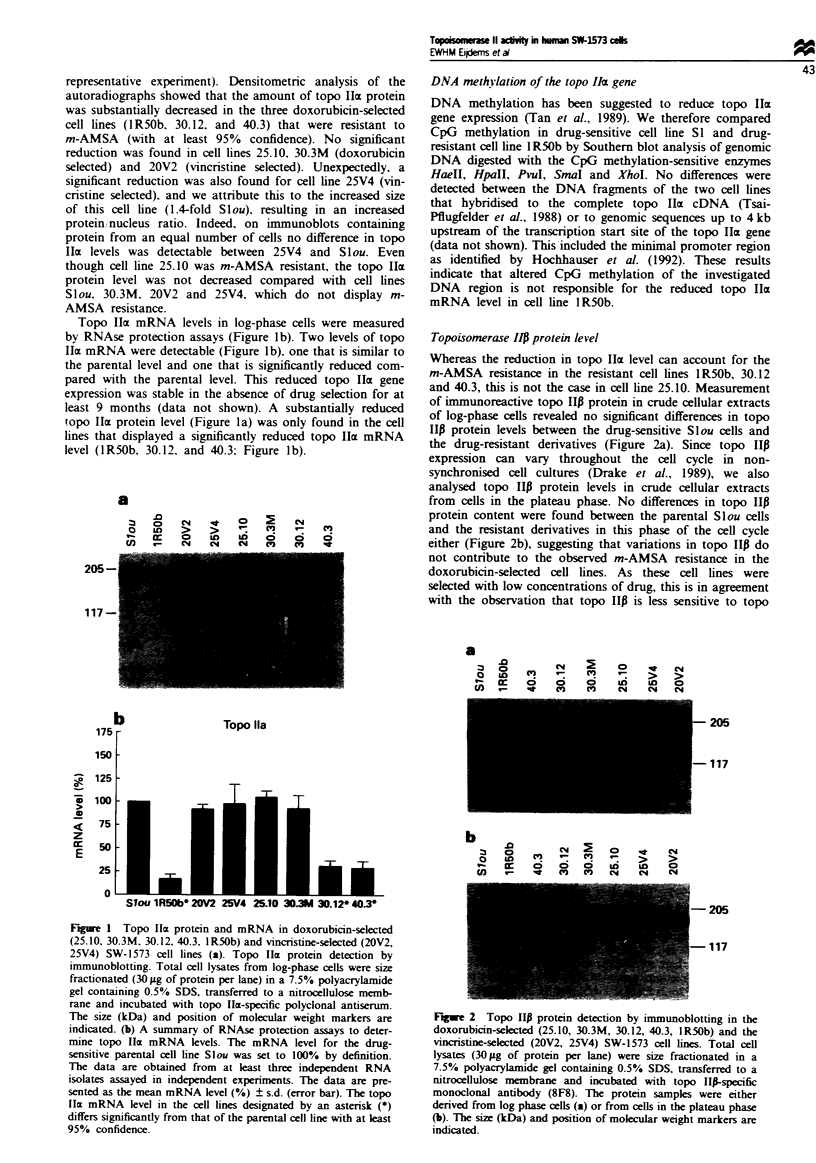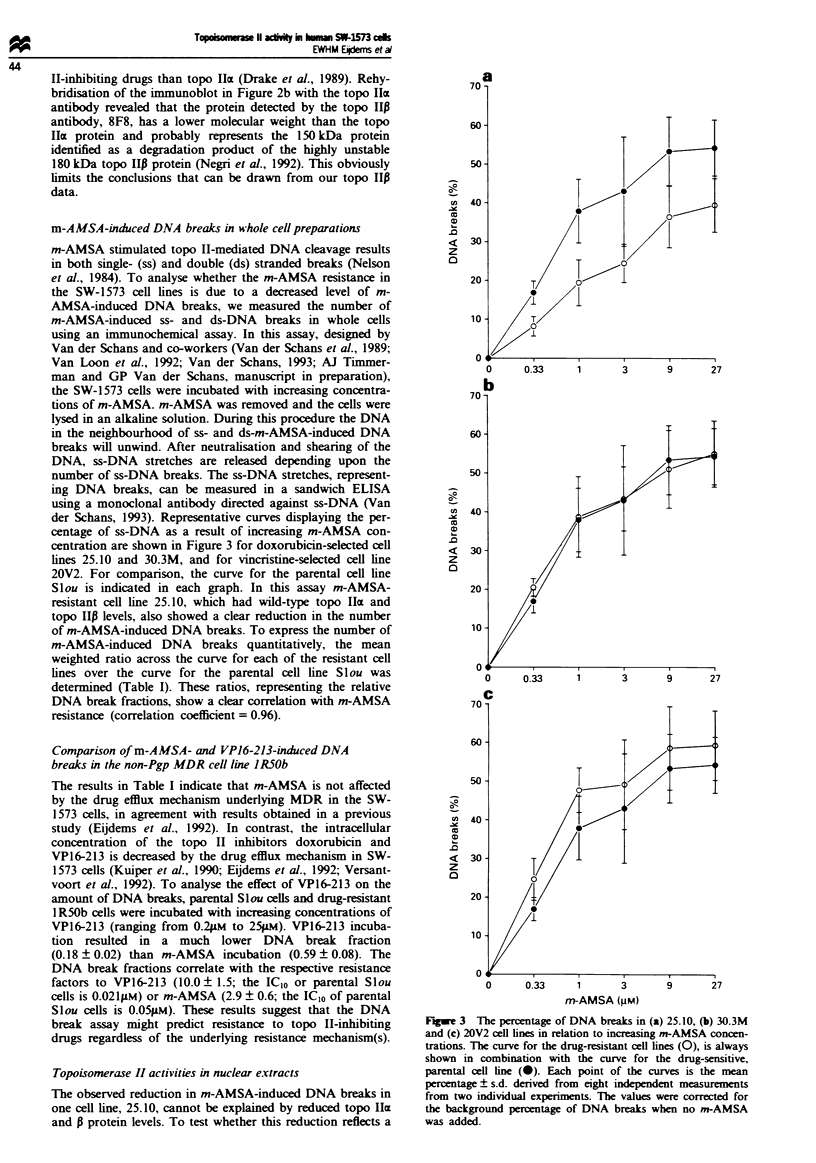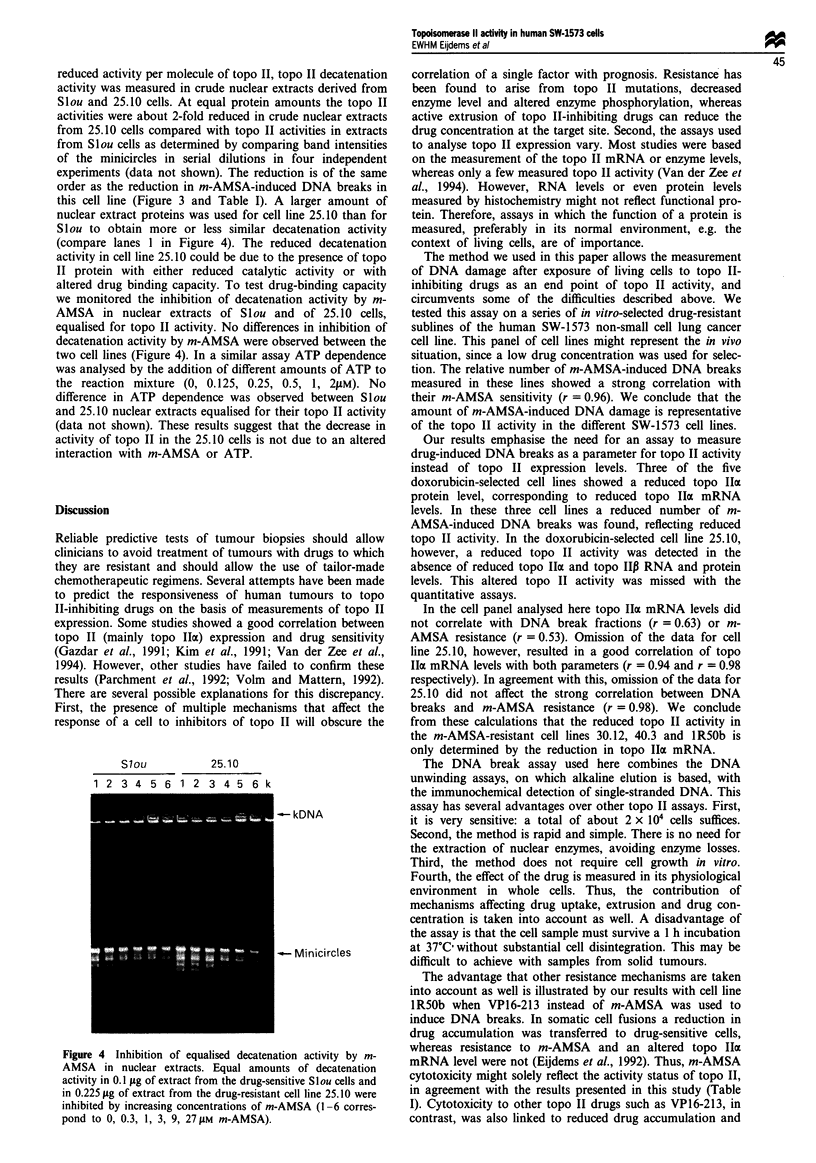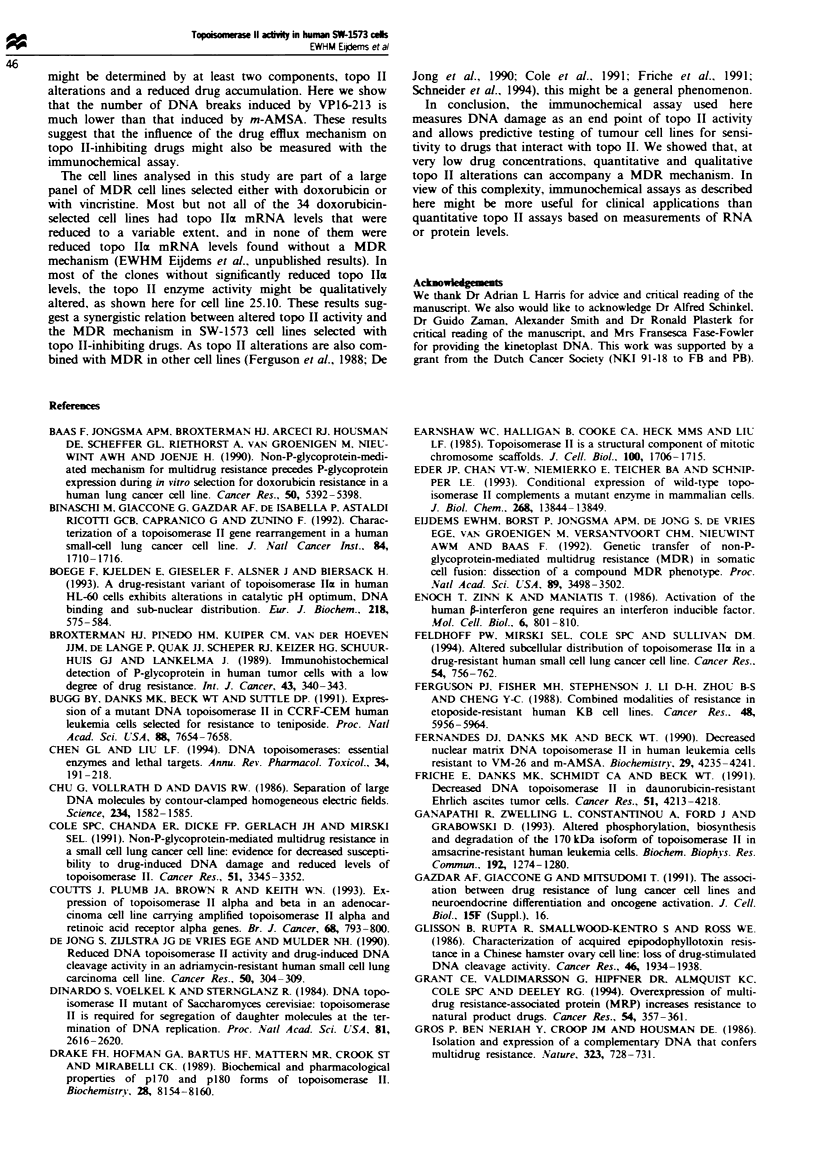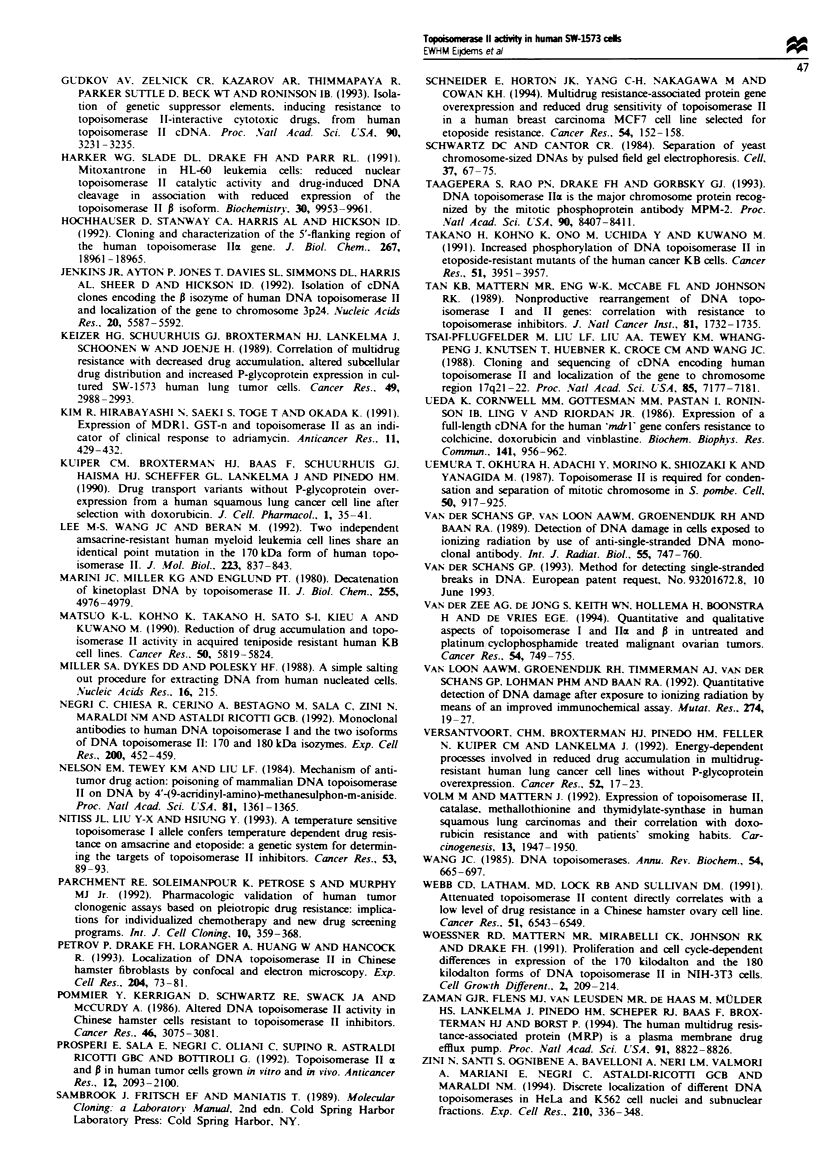# Reduced topoisomerase II activity in multidrug-resistant human non-small cell lung cancer cell lines.

**DOI:** 10.1038/bjc.1995.9

**Published:** 1995-01

**Authors:** E. W. Eijdems, M. de Haas, A. J. Timmerman, G. P. Van der Schans, E. Kamst, J. de Nooij, G. C. Astaldi Ricotti, P. Borst, F. Baas

**Affiliations:** Division of Molecular Biology, The Netherlands Cancer Institute, Amsterdam.

## Abstract

**Images:**


British Journal of Cancer (1995) 71, 40–47

© 1995 Stockton Press All rights reserved 0007–0920/95 $9.00

### Reduced topoisomerase II activity in multidrug-resistant human non-small cell lung cancer cell lines

EWHM Eijdems[1], M de Haas[1], AJ Timmerman[3], GP Van der Schans[3], E Kamst[1], J de Nooij[1], GCB Astaldi Ricotti[4], P Borst[1] and F Baas[1,2]

[1]*Division of Molecular Biology, The Netherlands Cancer Institute, Plesmanlaan 121, 1066 CX Amsterdam, The Netherlands;* [2]*Division of Neurology, Academical Medical Centre, 1105 AZ Amsterdam, The Netherlands;* [3]*TNO Nutrition and Food Research Institute, 2280 HV Rijswijk, The Netherlands;* [4]*Instituto di Genetica ed Evoluzionistica CNR, 27100 Pavia, Italy.*

**Summary** Multidrug-resistant (MDR) cell lines often have a compound phenotype, combining reduced drug accumulation with a decrease in topoisomerase II. We have analysed alterations in topoisomerase II in MDR derivatives of the human lung cancer cell line SW-1573. Selection with doxorubicin frequently resulted in reduced topo IIα mRNA and protein levels, whereas clones selected with vincristine showed normal levels of topo IIα. No alterations of topo IIβ levels were detected. To determine the contribution of topo II alterations to drug resistance, topo II activity was analysed by the determination of DNA breaks induced by the topo II-inhibiting drug 4'-(9-acridinylamino)methane-sulphon-*m*-anisidide (*m*-AMSA) in living cells, as *m*-AMSA is not affected by the drug efflux mechanism in the SW-1573 cells. The number of *m*-AMSA-induced DNA breaks correlated well ($r = 0.96$) with *in vitro* *m*-AMSA sensitivity. Drug sensitivity, however, did not always correlate with reduced topo II mRNA or protein levels. In one of the five doxorubicin-selected clones *m*-AMSA resistance and a reduction in *m*-AMSA-induced DNA breaks were found in the absence of reduced topo II protein levels. Therefore, we assume that post-translational modifications of topo II also contribute to drug resistance in SW-1573 cells. These results suggest that methods that detect quantitative as well as qualitative alterations of topo II should be used to predict the responsiveness of tumours to cytotoxic agents. The assay we used, which measures DNA breaks as an end point of topo II activity, could be a good candidate.

**Keywords:** topoisomerase II; multidrug resistance; P-glycoprotein; DNA breaks; *m*-AMSA

The occurrence of resistance to chemotherapeutic drugs contributes to the incurability of tumours and is a major problem for successful cancer treatment. In view of the major side-effects of chemotherapy, treatment with drugs to which the tumour cells display an intrinsic or acquired resistance should be avoided. Assays to predict the responsiveness to chemotherapeutic drugs are therefore of great interest for successful chemotherapeutic treatment of tumours. Drugs, such as doxorubicin, etoposide and 4'-(9-acridinylamino) methane-sulphon-*m*-anisidide (*m*-AMSA), which inhibit the enzyme topoisomerase II (topo II) are widely used as anti-tumour agents. Currently, two main categories of resistance mechanisms are known to interfere with this kind of drug. The first category concerns mechanisms resulting in a reduced drug concentration at the target site and will give a multidrug resistance (MDR) phenotype. Two drug efflux pumps have been identified that can cause a reduced drug concentration at the target site, P-glycoprotein (Gros *et al.*, 1986; Ueda *et al.*, 1986) and the multidrug resistance-associated protein (MRP) (Grant *et al.*, 1994; Zaman *et al.*, 1994). The second category of resistance, on which we will focus in this study, involves alterations affecting the drug target, the enzyme topo II.

Topo II is an ubiquitous enzyme that can alter the topological state of DNA and untangle intertwined DNA helices (reviewed in Chen and Liu, 1994; Wang, 1985). As such, it plays an essential role in several cellular events such as replication (DiNardo *et al.*, 1984), chromatin condensation (Uemura *et al.*, 1987) and sister chromatid segregation (DiNardo *et al.*, 1984; Uemura *et al.*, 1987). Topo II is also one of the major components of the nuclear matrix and the chromosomal scaffold (Earnshaw *et al.*, 1985; Taagepera *et al.*, 1993). Topo II cleaves double-stranded DNA and binds covalently to both strands of the molecule in the process. The transient reaction intermediate is called the 'cleavable complex'. Upon binding of ATP, a second DNA helix can pass through the cleavable complex, followed by hydrolysis of ATP and resealing of the double-stranded DNA (Chen and Liu, 1994). Topo II-inhibiting drugs stabilise the cleavable complex, resulting in both single- and double-strand DNA breaks (Nelson *et al.*, 1984; Glisson *et al.*, 1986), which can lead to cell death.

Two topo II isoforms, topo IIα (170 kDa) and topo IIβ (180 kDa), exist in animal cells. Although both enzymes are closely related (72% identical amino acid residues; Jenkins *et al.*, 1992), they differ in important biochemical and biophysical properties. It has been reported for some human cell lines that the topo IIα form is predominantly located in the nucleoplasm (Zini *et al.*, 1994), whereas the topo IIβ form is mainly located in the nucleolus (Boege *et al.*, 1993; Coutts *et al.*, 1993; Zini *et al.*, 1994). In Chinese hamster ovary cells both isoforms are detected in the nucleoplasm and in the nucleolus (Petrov *et al.*, 1993). Topo IIα expression is low in quiescent cells, increases in S-phase and is maximal in $G_2$–M phase, whereas topo IIβ expression is constant throughout the cell cycle (Woessner *et al.*, 1991; Prosperi *et al.*, 1992). Topo IIα is more sensitive to topo II-inhibiting drugs than topo IIβ and is thus more susceptible to the formation of a drug-stabilised cleavable complex (Drake *et al.*, 1989).

Resistance to topo II-inhibiting drugs can result from any process that results in an altered binding of topo II to drugs or DNA and thus a reduction in the number of cleavable complexes. Recent *in vivo* studies have provided direct evidence that a reduction in the topo IIα level results in resistance to topo II-inhibiting drugs such as etoposide and *m*-AMSA (Eder *et al.*, 1993; Gudkov *et al.*, 1993; Nitiss *et al.*, 1993). As different sensitivities of the topo IIα and β isoforms to topo II-inhibiting drugs have been reported (Drake *et al.*, 1989), the relative amount of each isozyme may also be a determinant of resistance to anti-tumour drugs.

In cell lines selected for resistance to topo II-inhibiting drugs, the topo IIα enzyme levels may be reduced (De Jong *et al.*, 1990; Matsuo *et al.*, 1990; Cole *et al.*, 1991; Friche *et al.*, 1991; Webb *et al.*, 1991; Eijdems *et al.*, 1992), the enzyme may be altered (Fernandes *et al.*, 1990; Bugg *et al.*, 1991; Binaschi *et al.*, 1992; Lee *et al.*, 1992; Schneider *et al.*, 1994),

Correspondence: P Borst
Received 12 July 1994; revised 25 August 1994; accepted 26 August 1994

or both reduced levels and alteration (Feldhoff *et al.*, 1994) may play a role in drug resistance. Since topo IIβ was identified only recently, most studies have focused on topo IIα. In one study, however, reduced amounts of the topo IIβ protein were associated with reduced topo II activity and increased drug resistance (Harker *et al.*, 1991). Post-translational modifications of topo IIα also appear to affect the catalytic activity and sensitivity to drugs in resistant cell lines (Takano *et al.*, 1991; Boege *et al.*, 1993; Ganapathi *et al.*, 1993).

We have previously shown that in doxorubicin-selected variants of the human SW-1573 non-small cell lung cancer cell line drug resistance is due to two alterations: a decreased drug accumulation and a reduced topo IIα mRNA level (Eijdems *et al.*, 1992). The drug accumulation defect and the alteration in topo IIα mRNA are not genetically linked and could be separated by somatic cell fusion (Eijdems *et al.*, 1992). The resulting MDR hybrid cells with a drug accumulation defect had normal topo IIα mRNA levels and had lost resistance to *m*-AMSA. The MDR hybrids were still partially resistant to doxorubicin and etoposide, most probably because of the drug accumulation defect. Subsequent analysis of a large set of MDR SW-1573 cell lines, selected with either doxorubicin or vincristine, revealed that the topo IIα mRNA levels were only decreased in the doxorubicin-selected cell lines (EWHM Eijdems *et al.*, unpublished data). This demonstrated that a reduction of topo IIα mRNA levels frequently accompanied a MDR mechanism when these lung cancer cell lines were selected for resistance against topo II-inhibiting drugs.

Several clinical studies indicate a role for altered topo IIα levels in drug sensitivity of tumours (Gazdar *et al.*, 1991; Kim *et al.*, 1991; Van der Zee *et al.*, 1994). The prediction of sensitivity to topo II-inhibiting drugs did not always correspond with topo II expression levels however (Parchment *et al.*, 1992; Volm and Mattern, 1992). In this study, we analysed topo II levels (topo IIα and topo IIβ) and topo II activity in a subset of the SW-1573 cell lines selected for low-level doxorubicin or vincristine resistance. Our results show that the number of DNA breaks as a measure of topo II activity closely correlates with drug resistance, in contrast to measurement of topo II expression levels. Functional assays of topo II activity may therefore be essential for a reliable prediction of the drug sensitivity of tumours.

### Materials and methods

### *Chemicals*

Doxorubicin (doxorubicin hydrochloride) and ouabain were purchased from Sigma (St Louis, MO, USA), 4′-(9-acridinylamino)methanesulphon-*m*-anisidide(*m*-AMSA) from Parke-Davis (Warner-Lambert, Amsterdam, The Netherlands) and VP16-213 from Bristol Meyers (Bergisch Gladbach, Germany).

### *Cell lines*

The resistant cell lines described in this study were derived from the human non-small cell lung cancer cell line SW-1573, originally isolated and characterised by Dr A Leibovitz (Scott and White Clinic, Temple, TX, USA). Cell line S1*ou* is a subclone of the drug-sensitive parental cell line S1 (a SW-1573 subline which was morphologically homogeneous) transfected with the α-1 subunit of the murine sodium/potassium exchanger (Eijdems *et al.*, 1992). The non-Pgp MDR cell line 1R50b was isolated from cell line S1 by a multistep doxorubicin selection up to 50 nM (Broxterman *et al.*, 1989; Keizer *et al.*, 1989; Baas *et al.*, 1990). The other resistant cell lines were isolated as a single-cell clone from cell line S1*ou* in a single-step selection at 25 nM, 30 nM and at 40 nM doxorubicin or at 20 nM and at 25 nM vincristine (Eijdems *et al.*, unpublished data). For nomenclature we took the drug concentration (nM) used for selection followed by an unique clone number preceded by a 'V' in case of vincristine selection. The cells were grown as monolayers in Ham's F-10 medium (Gibco, Paisley, UK) supplemented with 10% fetal calf serum, 2 mM glutamine, penicillin (50 units $ml^{-1}$) and streptomycin (50 μg $ml^{-1}$). Cells were maintained in humidified air/5% carbon dioxide at 37°C. All cells were free of *Mycoplasma* as tested by the use of the Gen-Probe rapid *Mycoplasma* detection system (Gen-Probe, San Diego, CA, USA).

### *Assay of drug resistance*

The clonogenic survival assay was carried out as follows. In 24-well dishes 80 cells per well were plated (tissue culture cluster 3424, Costar, Cambridge, MA, USA) and allowed to attach to the wells. Cells were then grown for 8 days in the continuous presence of drug, fixed and stained with 0.2% crystal violet (Merck 820603) in 3.7% glutaraldehyde. The percentage of cells that were able to produce a colony of 50 cells was used as a measure of cell survival. The resistance was calculated as the ratio of $IC_{10}$ (inhibitory concentration at which 10% of the cells survive) of the resistant cell line to the $IC_{10}$ of the parental cell line.

### *Sodium dodecyl sulphate–polyacrylamide gel electrophoresis (SDS–PAGE) and Western blotting*

Crude cell lysates were made by lysis of cells in 10 mM potassium chloride, 1.5 mM magnesium chloride, 10 mM Tris–HCl pH 7.4 and 0.5% SDS supplemented with 1 mM phenylmethylsulphonyl fluoride (PMSF), leupeptide (2 μg $ml^{-1}$), pepstatin (1 μg $ml^{-1}$) and aprotinin (2 μg $ml^{-1}$). DNA was sheared by sonication. The protein concentrations were measured using the Bio-Rad protein assay (Bio-Rad, Richmond, CA, USA). A 30 μg protein sample was boiled for 3 min in sample buffer (65 mM Tris–HCl pH 6.8, 2.5% SDS, 5% glycerol, 5% 2-mercaptoethanol) and separated on a 7.5% polyacrylamide gel containing 0.1% SDS and transferred onto nitrocellulose paper (Schleicher & Schuell, Dassel, Germany) by electroblotting. The blots were probed with either the rabbit polyclonal anti-topo IIα antibody, raised against a synthetic topo IIα peptide (Cambridge Research Biochemicals, Cheshire, UK; No. OA-11-752), or the monoclonal anti-topo IIβ antibody, 8F8, raised against topo IIβ protein purified from HeLa cells (Negri *et al.*, 1992). Bound antibody was visualised with horseradish peroxidase-conjugated horse anti-rabbit IgG (CLB, Amsterdam, The Netherlands; No. PK17E) in case of topo IIα and with horseradish peroxidase-conjugated rabbit anti-mouse-IgG (Dako, Copenhagen, Denmark) in case of topo IIβ, both followed by enhanced chemiluminescence detection (Amersham, UK).

### *RNAse protection assay*

The RNAse protection assay was performed as described previously (Baas *et al.*, 1990). Ten micrograms of total cytoplasmic RNA from each cell line was used or 10 μg of *E. coli* tRNA (Boehringer Mannheim, Germany) as a negative control. The protected probes were visualised by electrophoresis through a denaturing 6% acrylamide gel containing 8 M urea, followed by autoradiography. The topo IIα probe was a 174 nt topo IIα cDNA fragment (nucleotide positions 1343–1517; Tsai-Plugfelder *et al.*, 1988). In all experiments a probe for γ-actin (Enoch *et al.*, 1986) was included as internal control.

### *Southern hybridisations*

To prepare Southern blots, genomic DNA was isolated as previously described (Miller *et al.*, 1988) or cells were embedded in low melting point agarose plugs (Gibco BRL, Gaithersburg, MD, USA), lysed and deproteinised as described previously (Schwartz and Cantor, 1984). After digestion the generated fragments were separated on a 1% agarose gel in a regular electric field or in a contour-clamped gel in a regular electric field or in a contour-clamped homogeneous electric field (CHEF) box and transferred to a nitrocellulose membrane as described by Sambrook et al. (1989). The DNA was hybridised with the following $^{32}$P-labelled probes: the complete human topo IIα cDNA hTOP2 (Tsai-Pflugfelder et al., 1988), and genomic clones derived from a yeast artificial chromosome (YAC) clone isolated from the CEPH YAC library (Chu et al., 1986), using two oligonucleotide primer sets corresponding to 5′ and 3′ topo IIα cDNA sequences, and cloned into Bluescript M13 (Promega, Madison, WI, USA).

*Detection of* m-*AMSA-induced DNA breaks*

The detection of *m*-AMSA-induced DNA breaks is based on an immunochemical method developed for the detection of (radiation-induced) single-stranded regions in cellular DNA (Van der Schans et al., 1989; Van Loon et al., 1992). Cells in the log phase were incubated in Ham's F-10 medium containing increasing concentrations of *m*-AMSA for 1 h at 37°C. Cells were then washed twice in ice-cold phosphate buffered saline (PBS), scraped in ice-cold Ham's F-10 medium at a concentration of $0.5 \times 10^6$ cells ml$^{-1}$ and maintained on ice until assayed. DNA was denatured by the addition of 200 μl of alkaline solution A (1.3 M sodium chloride, adjusted with sodium hydroxide to pH 12.3) to 30 μl of cell suspension ($1.5 \times 10^4$ cells). After 6 min the solution was neutralised with 35 μl of 250 mM sodium dihydrogen phosphate, followed by fragmentation of the DNA by sonication (Ultrasonics W-370, USA, output level 2.5) for 1 s at 20°C to prevent annealing. After this treatment the DNA consisted of double- and single-stranded fragments. The fraction of single-stranded DNA was determined with a sandwich enzyme-linked immunosorbent assay (ELISA) that will be described in detail elsewhere (AJ Timmerman and GP Van der Schans, manuscript in preparation). In this sandwich ELISA, a modification of the earlier described ELISA (Van der Schans et al., 1989; Van Loon et al., 1992), the single-stranded DNA fragments were quantitatively attached to a polystyrene microtitre plate (Costar 9018) coated with monoclonal antibody specific for single-stranded DNA (Van der Schans, 1993). Subsequently, an excess of monoclonal antibody directed against single-stranded DNA conjugated to the enzyme alkaline phosphatase was added. The amount of antibody bound was determined by the measurement of fluorescence of 4-methylumbellipheryl phosphate converted into the fluorescent 4-methylumbelliferon by alkaline phosphatase. The relative amount of single-stranded DNA was determined by comparison with a sample of the same material that was made completely single stranded. The amount of the single-stranded DNA on the plate is a direct measure of single-strandedness, and hence the number of single-stranded DNA breaks.

*Preparation of nuclear enzyme extracts*

Nuclei were isolated as described previously (Pommier et al., 1986). Briefly, log-phase cells ($2.5 \times 10^5$ cells ml$^{-1}$) were pelleted by centrifugation at 150 *g* for 10 min and washed twice with ice-cold PBS. The cell pellets were resuspended in 1 ml of nucleus buffer (150 mM sodium chloride, 1 mM potassium dihydrogen phosphate, 5 mM magnesium chloride, 1 mM EGTA, 2 mM dithiotreitol and 1 mM PMSF, pH 6.4) at 4°C and then mixed with an additional 9 ml of nucleus buffer containing 0.3% Triton X-100. The cell suspension was mixed gently by rotation for 10 min at 4°C followed by centrifugation at 150 *g* for 10 min at 4°C. The nuclear pellet was washed once with Triton-free nucleus buffer, centrifuged and nuclei were extracted in 200 μl of nucleus buffer containing 0.35 M sodium chloride (final concentration) for 30 min at 4°C. The protein concentrations were determined using the Bio-Rad protein assay. The enzyme solution was diluted with an equal volume of 87% glycerol and stored at − 70°C.

*Decatenation assay*

The decatenation assay was carried out as previously described (Marini et al., 1980). The standard reaction mixture for the decatenation assay was 50 mM Tris-HCl (pH 7.5), 85 mM potassium chloride, 10 mM magnesium chloride, 0.5 mM dithiothreitol, 0.5 mM EDTA, bovine serum albumin (0.03 mg ml$^{-1}$) and 1 mM ATP. Decatenation of form I kinetoplast DNA (kDNA) from *C. fasciculata* was carried out by incubation of 5 μl of nuclear extract with 0.3 μg of kDNA in a final reaction volume of 25 μl of standard reaction mixture for 30 min at 37°C. Reactions were terminated by the addition of 5 μl of 3% SDS, 0.3% bromophenol blue and 30% glycerol. Samples were assayed by electrophoresis in 1% agarose in 89 mM Tris-borate, 2 mM EDTA (pH 8.3) at 3.7 V cm$^{-1}$ for 4 h. Gels were stained in ethidium bromide (1 μg ml$^{-1}$), destained and photographed under UV light.

### Results

*Sensitivity to topoisomerase II-inhibiting drugs*

In previous studies we generated MDR SW-1573 sublines by selection with either doxorubicin or vincristine. Nearly all cell lines had a reduced drug accumulation without overexpression of P-glycoprotein. Alterations in MRP seemed to be involved in their MDR phenotype and a considerable overexpression of *MRP* was detectable in one of them, cell line 30.3M (EWHM Eijdems et al., unpublished results). To study the contribution of topo II alterations to drug resistance, the sensitivity to the topo II-inhibiting drugs doxorubicin and *m*-AMSA was determined in seven of these cell lines (Table I). All sublines were resistant to doxorubicin, but only the doxorubicin-selected cell lines, with the exception of 30.3M, were resistant to *m*-AMSA. The vincristine-selected sublines, 20V2 and 25V4, even displayed a slightly increased sensitivity to *m*-AMSA compared with the parental cell line S1*ou*. These results suggest that *m*-AMSA is not affected by the drug efflux mechanism present in these SW-1573 cell lines and might therefore be a suitable drug to study the effect of drug selection on topo II activity.

*Topoisomerase IIα mRNA and protein level*

As reduced topo IIα levels could be responsible for the observed *m*-AMSA resistance, we determined immunoreactive topo IIα levels in cellular extracts of log-phase SW-1573 cells in four independent experiments (see Figure 1a for a

**Table I** Drug resistance to topoisomerase II-inhibiting drugs and the *m*-AMSA-induced DNA break fraction relative to the parental cells in doxorubicin- and vincristine-selected SW-1573 sublines

| | *Resistance factor*[a,b] | | *Relative DNA* |
|---|---|---|---|
| | *Doxorubicin* | m-*AMSA* | *break fraction*[c] |
| *Doxorubicin-selected* | | | |
| 25.10 | 2.3 ± 0.7 | 3.3 ± 0.6 | 0.60 ± 0.08 |
| 30.3M | 3.7 ± 1.2 | 1.0 ± 0.1 | 1.04 ± 0.12 |
| 30.12 | 3.1 ± 0.9 | 2.2 ± 0.3 | 0.70 ± 0.09 |
| 40.3 | 4.8 ± 1.5 | 2.5 ± 0.3 | 0.69 ± 0.07 |
| 1R50b | 7.0 ± 1.2 | 2.9 ± 0.6 | 0.59 ± 0.08 |
| *Vincristine-selected* | | | |
| 20V2 | 3.1 ± 0.8 | 0.8 ± 0.2 | 1.19 ± 0.13 |
| 25V4 | 2.9 ± 0.7 | 0.7 ± 0.1 | 1.11 ± 0.14 |

[a]The resistance factor is expressed as the ratio of the $IC_{10}$ of the resistant subline to the $IC_{10}$ of the parental cell line S1*ou* ($IC_{10}$ = inhibitory concentration at which 10% of the cells survive). The $IC_{10}$ of cell line S1*ou* is for doxorubicin 0.015 μM and for *m*-AMSA 0.050 μM. [b]The data are presented as the mean resistance factor ± s.d. of at least three independent experiments each performed in duplicate. [c]The values for the *m*-AMSA-induced DNA break fraction are presented as the weighted ratios of the points of the curve for the drug-resistant cell line over that of the parental cell line S1*ou*. All points are the mean of eight independent measurements obtained in two individual experiments.

representative experiment). Densitometric analysis of the autoradiographs showed that the amount of topo IIα protein was substantially decreased in the three doxorubicin-selected cell lines (1R50b, 30.12, and 40.3) that were resistant to *m*-AMSA (with at least 95% confidence). No significant reduction was found in cell lines 25.10, 30.3M (doxorubicin selected) and 20V2 (vincristine selected). Unexpectedly, a significant reduction was also found for cell line 25V4 (vincristine selected), and we attribute this to the increased size of this cell line (1.4-fold S1*ou*), resulting in an increased protein/nucleus ratio. Indeed, on immunoblots containing protein from an equal number of cells no difference in topo IIα levels was detectable between 25V4 and S1*ou*. Even though cell line 25.10 was *m*-AMSA resistant, the topo IIα protein level was not decreased compared with cell lines S1*ou*, 30.3M, 20V2 and 25V4, which do not display *m*-AMSA resistance.

Topo IIα mRNA levels in log-phase cells were measured by RNAse protection assays (Figure 1b). Two levels of topo IIα mRNA were detectable (Figure 1b), one that is similar to the parental level and one that is significantly reduced compared with the parental level. This reduced topo IIα gene expression was stable in the absence of drug selection for at least 9 months (data not shown). A substantially reduced topo IIα protein level (Figure 1a) was only found in the cell lines that displayed a significantly reduced topo IIα mRNA level (1R50b, 30.12, and 40.3; Figure 1b).

**Figure 1** Topo IIα protein and mRNA in doxorubicin-selected (25.10, 30.3M, 30.12, 40.3, 1R50b) and vincristine-selected (20V2, 25V4) SW-1573 cell lines (**a**). Topo IIα protein detection by immunoblotting. Total cell lysates from log-phase cells were size fractionated (30 μg of protein per lane) in a 7.5% polyacrylamide gel containing 0.5% SDS, transferred to a nitrocellulose membrane and incubated with topo IIα-specific polyclonal antiserum. The size (kDa) and position of molecular weight markers are indicated. (**b**) A summary of RNAse protection assays to determine topo IIα mRNA levels. The mRNA level for the drug-sensitive parental cell line S1*ou* was set to 100% by definition. The data are obtained from at least three independent RNA isolates assayed in independent experiments. The data are presented as the mean mRNA level (%) ± s.d. (error bar). The topo IIα mRNA level in the cell lines designated by an asterisk (*) differs significantly from that of the parental cell line with at least 95% confidence.

*DNA methylation of the topo IIα gene*

DNA methylation has been suggested to reduce topo IIα gene expression (Tan *et al.*, 1989). We therefore compared CpG methylation in drug-sensitive cell line S1 and drug-resistant cell line 1R50b by Southern blot analysis of genomic DNA digested with the CpG methylation-sensitive enzymes *Hae*II, *Hpa*II, *Pvu*I, *Sma*I and *Xho*I. No differences were detected between the DNA fragments of the two cell lines that hybridised to the complete topo IIα cDNA (Tsai-Pflugfelder *et al.*, 1988) or to genomic sequences up to 4 kb upstream of the transcription start site of the topo IIα gene (data not shown). This included the minimal promoter region as identified by Hochhauser *et al.* (1992). These results indicate that altered CpG methylation of the investigated DNA region is not responsible for the reduced topo IIα mRNA level in cell line 1R50b.

*Topoisomerase IIβ protein level*

Whereas the reduction in topo IIα level can account for the *m*-AMSA resistance in the resistant cell lines 1R50b, 30.12 and 40.3, this is not the case in cell line 25.10. Measurement of immunoreactive topo IIβ protein in crude cellular extracts of log-phase cells revealed no significant differences in topo IIβ protein levels between the drug-sensitive S1*ou* cells and the drug-resistant derivatives (Figure 2a). Since topo IIβ expression can vary throughout the cell cycle in non-synchronised cell cultures (Drake *et al.*, 1989), we also analysed topo IIβ protein levels in crude cellular extracts from cells in the plateau phase. No differences in topo IIβ protein content were found between the parental S1*ou* cells and the resistant derivatives in this phase of the cell cycle either (Figure 2b), suggesting that variations in topo IIβ do not contribute to the observed *m*-AMSA resistance in the doxorubicin-selected cell lines. As these cell lines were selected with low concentrations of drug, this is in agreement with the observation that topo IIβ is less sensitive to topo

**Figure 2** Topo IIβ protein detection by immunoblotting in the doxorubicin-selected (25.10, 30.3M, 30.12, 40.3, 1R50b) and the vincristine-selected (20V2, 25V4) SW-1573 cell lines. Total cell lysates (30 μg of protein per lane) were size fractionated in a 7.5% polyacrylamide gel containing 0.5% SDS, transferred to a nitrocellulose membrane and incubated with topo IIβ-specific monoclonal antibody (8F8). The protein samples were either derived from log phase cells (**a**) or from cells in the plateau phase (**b**). The size (kDa) and position of molecular weight markers are indicated.

II-inhibiting drugs than topo IIα (Drake *et al.*, 1989). Rehybridisation of the immunoblot in Figure 2b with the topo IIα antibody revealed that the protein detected by the topo IIβ antibody, 8F8, has a lower molecular weight than the topo IIα protein and probably represents the 150 kDa protein identified as a degradation product of the highly unstable 180 kDa topo IIβ protein (Negri *et al.*, 1992). This obviously limits the conclusions that can be drawn from our topo IIβ data.

### m-*AMSA-induced DNA breaks in whole cell preparations*

*m*-AMSA stimulated topo II-mediated DNA cleavage results in both single- (ss) and double (ds) stranded breaks (Nelson *et al.*, 1984). To analyse whether the *m*-AMSA resistance in the SW-1573 cell lines is due to a decreased level of *m*-AMSA-induced DNA breaks, we measured the number of *m*-AMSA-induced ss- and ds-DNA breaks in whole cells using an immunochemical assay. In this assay, designed by Van der Schans and co-workers (Van der Schans *et al.*, 1989; Van Loon *et al.*, 1992; Van der Schans, 1993; AJ Timmerman and GP Van der Schans, manuscript in preparation), the SW-1573 cells were incubated with increasing concentrations of *m*-AMSA. *m*-AMSA was removed and the cells were lysed in an alkaline solution. During this procedure the DNA in the neighbourhood of ss- and ds-*m*-AMSA-induced DNA breaks will unwind. After neutralisation and shearing of the DNA, ss-DNA stretches are released depending upon the number of ss-DNA breaks. The ss-DNA stretches, representing DNA breaks, can be measured in a sandwich ELISA using a monoclonal antibody directed against ss-DNA (Van der Schans, 1993). Representative curves displaying the percentage of ss-DNA as a result of increasing *m*-AMSA concentration are shown in Figure 3 for doxorubicin-selected cell lines 25.10 and 30.3M, and for vincristine-selected cell line 20V2. For comparison, the curve for the parental cell line S1*ou* is indicated in each graph. In this assay *m*-AMSA-resistant cell line 25.10, which had wild-type topo IIα and topo IIβ levels, also showed a clear reduction in the number of *m*-AMSA-induced DNA breaks. To express the number of *m*-AMSA-induced DNA breaks quantitatively, the mean weighted ratio across the curve for each of the resistant cell lines over the curve for the parental cell line S1*ou* was determined (Table I). These ratios, representing the relative DNA break fractions, show a clear correlation with *m*-AMSA resistance (correlation coefficient = 0.96).

### *Comparison of* m-*AMSA- and VP16-213-induced DNA breaks in the non-Pgp MDR cell line 1R50b*

The results in Table I indicate that *m*-AMSA is not affected by the drug efflux mechanism underlying MDR in the SW-1573 cells, in agreement with results obtained in a previous study (Eijdems *et al.*, 1992). In contrast, the intracellular concentration of the topo II inhibitors doxorubicin and VP16-213 is decreased by the drug efflux mechanism in SW-1573 cells (Kuiper *et al.*, 1990; Eijdems *et al.*, 1992; Versantvoort *et al.*, 1992). To analyse the effect of VP16-213 on the amount of DNA breaks, parental S1*ou* cells and drug-resistant 1R50b cells were incubated with increasing concentrations of VP16-213 (ranging from 0.2μM to 25μM). VP16-213 incubation resulted in a much lower DNA break fraction (0.18 ± 0.02) than *m*-AMSA incubation (0.59 ± 0.08). The DNA break fractions correlate with the respective resistance factors to VP16-213 (10.0 ± 1.5; the $IC_{10}$ or parental S1*ou* cells is 0.021μM) or *m*-AMSA (2.9 ± 0.6; the $IC_{10}$ of parental S1*ou* cells is 0.05μM). These results suggest that the DNA break assay might predict resistance to topo II-inhibiting drugs regardless of the underlying resistance mechanism(s).

### *Topoisomerase II activities in nuclear extracts*

The observed reduction in *m*-AMSA-induced DNA breaks in one cell line, 25.10, cannot be explained by reduced topo IIα and β protein levels. To test whether this reduction reflects a

**Figure 3** The percentage of DNA breaks in (**a**) 25.10, (**b**) 30.3M and (**c**) 20V2 cell lines in relation to increasing *m*-AMSA concentrations. The curve for the drug-resistant cell lines (○), is always shown in combination with the curve for the drug-sensitive, parental cell line (●). Each point of the curves is the mean percentage ± s.d. derived from eight independent measurements from two individual experiments. The values were corrected for the background percentage of DNA breaks when no *m*-AMSA was added.

reduced activity per molecule of topo II, topo II decatenation activity was measured in crude nuclear extracts derived from S1*ou* and 25.10 cells. At equal protein amounts the topo II activities were about 2-fold reduced in crude nuclear extracts from 25.10 cells compared with topo II activities in extracts from S1*ou* cells as determined by comparing band intensities of the minicircles in serial dilutions in four independent experiments (data not shown). The reduction is of the same order as the reduction in *m*-AMSA-induced DNA breaks in this cell line (Figure 3 and Table I). A larger amount of nuclear extract proteins was used for cell line 25.10 than for S1*ou* to obtain more or less similar decatenation activity (compare lanes 1 in Figure 4). The reduced decatenation activity in cell line 25.10 could be due to the presence of topo II protein with either reduced catalytic activity or with altered drug binding capacity. To test drug-binding capacity we monitored the inhibition of decatenation activity by *m*-AMSA in nuclear extracts of S1*ou* and of 25.10 cells, equalised for topo II activity. No differences in inhibition of decatenation activity by *m*-AMSA were observed between the two cell lines (Figure 4). In a similar assay ATP dependence was analysed by the addition of different amounts of ATP to the reaction mixture (0, 0.125, 0.25, 0.5, 1, 2μM). No difference in ATP dependence was observed between S1*ou* and 25.10 nuclear extracts equalised for their topo II activity (data not shown). These results suggest that the decrease in activity of topo II in the 25.10 cells is not due to an altered interaction with *m*-AMSA or ATP.

### Discussion

Reliable predictive tests of tumour biopsies should allow clinicians to avoid treatment of tumours with drugs to which they are resistant and should allow the use of tailor-made chemotherapeutic regimens. Several attempts have been made to predict the responsiveness of human tumours to topo II-inhibiting drugs on the basis of measurements of topo II expression. Some studies showed a good correlation between topo II (mainly topo IIα) expression and drug sensitivity (Gazdar *et al.*, 1991; Kim *et al.*, 1991; Van der Zee *et al.*, 1994). However, other studies have failed to confirm these results (Parchment *et al.*, 1992; Volm and Mattern, 1992). There are several possible explanations for this discrepancy. First, the presence of multiple mechanisms that affect the response of a cell to inhibitors of topo II will obscure the correlation of a single factor with prognosis. Resistance has been found to arise from topo II mutations, decreased enzyme level and altered enzyme phosphorylation, whereas active extrusion of topo II-inhibiting drugs can reduce the drug concentration at the target site. Second, the assays used to analyse topo II expression vary. Most studies were based on the measurement of the topo II mRNA or enzyme levels, whereas only a few measured topo II activity (Van der Zee *et al.*, 1994). However, RNA levels or even protein levels measured by histochemistry might not reflect functional protein. Therefore, assays in which the function of a protein is measured, preferably in its normal environment, e.g. the context of living cells, are of importance.

**Figure 4** Inhibition of equalised decatenation activity by *m*-AMSA in nuclear extracts. Equal amounts of decatenation activity in 0.1 μg of extract from the drug-sensitive S1*ou* cells and in 0.225 μg of extract from the drug-resistant cell line 25.10 were inhibited by increasing concentrations of *m*-AMSA (1–6 correspond to 0, 0.3, 1, 3, 9, 27 μM *m*-AMSA).

The method we used in this paper allows the measurement of DNA damage after exposure of living cells to topo II-inhibiting drugs as an end point of topo II activity, and circumvents some of the difficulties described above. We tested this assay on a series of *in vitro*-selected drug-resistant sublines of the human SW-1573 non-small cell lung cancer cell line. This panel of cell lines might represent the *in vivo* situation, since a low drug concentration was used for selection. The relative number of *m*-AMSA-induced DNA breaks measured in these lines showed a strong correlation with their *m*-AMSA sensitivity ($r = 0.96$). We conclude that the amount of *m*-AMSA-induced DNA damage is representative of the topo II activity in the different SW-1573 cell lines.

Our results emphasise the need for an assay to measure drug-induced DNA breaks as a parameter for topo II activity instead of topo II expression levels. Three of the five doxorubicin-selected cell lines showed a reduced topo IIα protein level, corresponding to reduced topo IIα mRNA levels. In these three cell lines a reduced number of *m*-AMSA-induced DNA breaks was found, reflecting reduced topo II activity. In the doxorubicin-selected cell line 25.10, however, a reduced topo II activity was detected in the absence of reduced topo IIα and topo IIβ RNA and protein levels. This altered topo II activity was missed with the quantitative assays.

In the cell panel analysed here topo IIα mRNA levels did not correlate with DNA break fractions ($r = 0.63$) or *m*-AMSA resistance ($r = 0.53$). Omission of the data for cell line 25.10, however, resulted in a good correlation of topo IIα mRNA levels with both parameters ($r = 0.94$ and $r = 0.98$ respectively). In agreement with this, omission of the data for 25.10 did not affect the strong correlation between DNA breaks and *m*-AMSA resistance ($r = 0.98$). We conclude from these calculations that the reduced topo II activity in the *m*-AMSA-resistant cell lines 30.12, 40.3 and 1R50b is only determined by the reduction in topo IIα mRNA.

The DNA break assay used here combines the DNA unwinding assays, on which alkaline elution is based, with the immunochemical detection of single-stranded DNA. This assay has several advantages over other topo II assays. First, it is very sensitive: a total of about $2 \times 10^4$ cells suffices. Second, the method is rapid and simple. There is no need for the extraction of nuclear enzymes, avoiding enzyme losses. Third, the method does not require cell growth *in vitro*. Fourth, the effect of the drug is measured in its physiological environment in whole cells. Thus, the contribution of mechanisms affecting drug uptake, extrusion and drug concentration is taken into account as well. A disadvantage of the assay is that the cell sample must survive a 1 h incubation at 37°C without substantial cell disintegration. This may be difficult to achieve with samples from solid tumours.

The advantage that other resistance mechanisms are taken into account as well is illustrated by our results with cell line 1R50b when VP16-213 instead of *m*-AMSA was used to induce DNA breaks. In somatic cell fusions a reduction in drug accumulation was transferred to drug-sensitive cells, whereas resistance to *m*-AMSA and an altered topo IIα mRNA level were not (Eijdems *et al.*, 1992). Thus, *m*-AMSA cytotoxicity might solely reflect the activity status of topo II, in agreement with the results presented in this study (Table I). Cytotoxicity to other topo II drugs such as VP16-213, in contrast, was also linked to reduced drug accumulation and might be determined by at least two components, topo II alterations and a reduced drug accumulation. Here we show that the number of DNA breaks induced by VP16-213 is much lower than that induced by *m*-AMSA. These results suggest that the influence of the drug efflux mechanism on topo II-inhibiting drugs might also be measured with the immunochemical assay.

The cell lines analysed in this study are part of a large panel of MDR cell lines selected either with doxorubicin or with vincristine. Most but not all of the 34 doxorubicin-selected cell lines had topo IIα mRNA levels that were reduced to a variable extent, and in none of them were reduced topo IIα mRNA levels found without a MDR mechanism (EWHM Eijdems *et al*., unpublished results). In most of the clones without significantly reduced topo IIα levels, the topo II enzyme activity might be qualitatively altered, as shown here for cell line 25.10. These results suggest a synergistic relation between altered topo II activity and the MDR mechanism in SW-1573 cell lines selected with topo II-inhibiting drugs. As topo II alterations are also combined with MDR in other cell lines (Ferguson *et al*., 1988; De Jong *et al*., 1990; Cole *et al*., 1991; Friche *et al*., 1991; Schneider *et al*., 1994), this might be a general phenomenon.

In conclusion, the immunochemical assay used here measures DNA damage as an end point of topo II activity and allows predictive testing of tumour cell lines for sensitivity to drugs that interact with topo II. We showed that, at very low drug concentrations, quantitative and qualitative topo II alterations can accompany a MDR mechanism. In view of this complexity, immunochemical assays as described here might be more useful for clinical applications than quantitative topo II assays based on measurements of RNA or protein levels.

**Acknowledgements**

We thank Dr Adrian L Harris for advice and critical reading of the manuscript. We also would like to acknowledge Dr Alfred Schinkel, Dr Guido Zaman, Alexander Smith and Dr Ronald Plasterk for critical reading of the manuscript, and Mrs Fransesca Fase-Fowler for providing the kinetoplast DNA. This work was supported by a grant from the Dutch Cancer Society (NKI 91-18 to FB and PB).

**References**

BAAS F, JONGSMA APM, BROXTERMAN HJ, ARCECI RJ, HOUSMAN DE, SCHEFFER GL, RIETHORST A, VAN GROENIGEN M, NIEUWINT AWH AND JOENJE H. (1990). Non-P-glycoprotein-mediated mechanism for multidrug resistance precedes P-glycoprotein expression during *in vitro* selection for doxorubicin resistance in a human lung cancer cell line. *Cancer Res*., **50**, 5392–5398.

BINASCHI M, GIACCONE G, GAZDAR AF, DE ISABELLA P, ASTALDI RICOTTI GCB, CAPRANICO G AND ZUNINO F. (1992). Characterization of a topoisomerase II gene rearrangement in a human small-cell lung cancer cell line. *J. Natl Cancer Inst*., **84**, 1710–1716.

BOEGE F, KJELDEN E, GIESELER F, ALSNER J AND BIERSACK H. (1993). A drug-resistant variant of topoisomerase IIα in human HL-60 cells exhibits alterations in catalytic pH optimum, DNA binding and sub-nuclear distribution. *Eur. J. Biochem*., **218**, 575–584.

BROXTERMAN HJ, PINEDO HM, KUIPER CM, VAN DER HOEVEN JJM, DE LANGE P, QUAK JJ, SCHEPER RJ, KEIZER HG, SCHUURHUIS GJ AND LANKELMA J. (1989). Immunohistochemical detection of P-glycoprotein in human tumor cells with a low degree of drug resistance. *Int. J. Cancer*, **43**, 340–343.

BUGG BY, DANKS MK, BECK WT AND SUTTLE DP. (1991). Expression of a mutant DNA topoisomerase II in CCRF-CEM human leukemia cells selected for resistance to teniposide. *Proc. Natl Acad. Sci. USA*, **88**, 7654–7658.

CHEN GL AND LIU LF. (1994). DNA topoisomerases: essential enzymes and lethal targets. *Annu. Rev. Pharmacol. Toxicol*., **34**, 191–218.

CHU G, VOLLRATH D AND DAVIS RW. (1986). Separation of large DNA molecules by contour-clamped homogeneous electric fields. *Science*, **234**, 1582–1585.

COLE SPC, CHANDA ER, DICKE FP, GERLACH JH AND MIRSKI SEL. (1991). Non-P-glycoprotein-mediated multidrug resistance in a small cell lung cancer cell line: evidence for decreased susceptibility to drug-induced DNA damage and reduced levels of topoisomerase II. *Cancer Res*., **51**, 3345–3352.

COUTTS J, PLUMB JA, BROWN R AND KEITH WN. (1993). Expression of topoisomerase II alpha and beta in an adenocarcinoma cell line carrying amplified topoisomerase II alpha and retinoic acid receptor alpha genes. *Br. J. Cancer*, **68**, 793–800.

DE JONG S, ZIJLSTRA JG DE VRIES EGE AND MULDER NH. (1990). Reduced DNA topoisomerase II activity and drug-induced DNA cleavage activity in an adriamycin-resistant human small cell lung carcinoma cell line. *Cancer Res*., **50**, 304–309.

DINARDO S, VOELKEL K AND STERNGLANZ R. (1984). DNA topoisomerase II mutant of Saccharomyces cerevisiae: topoisomerase II is required for segregation of daughter molecules at the termination of DNA replication. *Proc. Natl Acad. Sci. USA*, **81**, 2616–2620.

DRAKE FH, HOFMAN GA, BARTUS HF, MATTERN MR, CROOK ST AND MIRABELLI CK. (1989). Biochemical and pharmacological properties of p170 and p180 forms of topoisomerase II. *Biochemistry*, **28**, 8154–8160.

EARNSHAW WC, HALLIGAN B, COOKE CA, HECK MMS AND LIU LF. (1985). Topoisomerase II is a structural component of mitotic chromosome scaffolds. *J. Cell. Biol*., **100**, 1706–1715.

EDER JP, CHAN VT-W, NIEMIERKO E, TEICHER BA AND SCHNIPPER LE. (1993). Conditional expression of wild-type topoisomerase II complements a mutant enzyme in mammalian cells. *J. Biol. Chem*., **268**, 13844–13849.

EIJDEMS EWHM, BORST P, JONGSMA APM, DE JONG S, DE VRIES EGE, VAN GROENIGEN M, VERSANTVOORT CHM, NIEUWINT AWM AND BAAS F. (1992). Genetic transfer of non-P-glycoprotein-mediated multidrug resistance (MDR) in somatic cell fusion: dissection of a compound MDR phenotype. *Proc. Natl Acad. Sci. USA*, **89**, 3498–3502.

ENOCH T, ZINN K AND MANIATIS T. (1986). Activation of the human β-interferon gene requires an interferon inducible factor. *Mol. Cell. Biol*., **6**, 801–810.

FELDHOFF PW, MIRSKI SEL, COLE SPC AND SULLIVAN DM. (1994). Altered subcellular distribution of topoisomerase IIα in a drug-resistant human small cell lung cancer cell line. *Cancer Res*., **54**, 756–762.

FERGUSON PJ, FISHER MH, STEPHENSON J, LI D-H, ZHOU B-S AND CHENG Y-C. (1988). Combined modalities of resistance in etoposide-resistant human KB cell lines. *Cancer Res*., **48**, 5956–5964.

FERNANDES DJ, DANKS MK AND BECK WT. (1990). Decreased nuclear matrix DNA topoisomerase II in human leukemia cells resistant to VM-26 and m-AMSA. *Biochemistry*, **29**, 4235–4241.

FRICHE E, DANKS MK, SCHMIDT CA AND BECK WT. (1991). Decreased DNA topoisomerase II in daunorubicin-resistant Ehrlich ascites tumor cells. *Cancer Res*., **51**, 4213–4218.

GANAPATHI R, ZWELLING L, CONSTANTINOU A, FORD J AND GRABOWSKI D. (1993). Altered phosphorylation, biosynthesis and degradation of the 170 kDa isoform of topoisomerase II in amsacrine-resistant human leukemia cells. *Biochem. Biophys. Res. Commun*., **192**, 1274–1280.

GAZDAR AF, GIACCONE G AND MITSUDOMI T. (1991). The association between drug resistance of lung cancer cell lines and neuroendocrine differentiation and oncogene activation. *J. Cell. Biol*., **15F** (Suppl.), 16.

GLISSON B, RUPTA R, SMALLWOOD-KENTRO S AND ROSS WE. (1986). Characterization of acquired epipodophyllotoxin resistance in a Chinese hamster ovary cell line: loss of drug-stimulated DNA cleavage activity. *Cancer Res*., **46**, 1934–1938.

GRANT CE, VALDIMARSSON G, HIPFNER DR, ALMQUIST KC, COLE SPC AND DEELEY RG. (1994). Overexpression of multidrug resistance-associated protein (MRP) increases resistance to natural product drugs. *Cancer Res*., **54**, 357–361.

GROS P, BEN NERIAH Y, CROOP JM AND HOUSMAN DE. (1986). Isolation and expression of a complementary DNA that confers multidrug resistance. *Nature*, **323**, 728–731.

GUDKOV AV, ZELNICK CR, KAZAROV AR, THIMMAPAYA R, PARKER SUTTLE D, BECK WT AND RONINSON IB. (1993). Isolation of genetic suppressor elements, inducing resistance to topoisomerase II-interactive cytotoxic drugs, from human topoisomerase II cDNA. *Proc. Natl Acad. Sci. USA*, **90**, 3231-3235.

HARKER WG, SLADE DL, DRAKE FH AND PARR RL. (1991). Mitoxantrone in HL-60 leukemia cells: reduced nuclear topoisomerase II catalytic activity and drug-induced DNA cleavage in association with reduced expression of the topoisomerase II β isoform. *Biochemistry*, **30**, 9953-9961.

HOCHHAUSER D, STANWAY CA, HARRIS AL AND HICKSON ID. (1992). Cloning and characterization of the 5'-flanking region of the human topoisomerase IIα gene. *J. Biol. Chem.*, **267**, 18961-18965.

JENKINS JR, AYTON P, JONES T, DAVIES SL, SIMMONS DL, HARRIS AL, SHEER D AND HICKSON ID. (1992). Isolation of cDNA clones encoding the β isozyme of human DNA topoisomerase II and localization of the gene to chromosome 3p24. *Nucleic Acids Res.*, **20**, 5587-5592.

KEIZER HG, SCHUURHUIS GJ, BROXTERMAN HJ, LANKELMA J, SCHOONEN W AND JOENJE H. (1989). Correlation of multidrug resistance with decreased drug accumulation, altered subcellular drug distribution and increased P-glycoprotein expression in cultured SW-1573 human lung tumor cells. *Cancer Res.*, **49**, 2988-2993.

KIM R, HIRABAYASHI N, SAEKI S, TOGE T AND OKADA K. (1991). Expression of MDR1, GST-π and topoisomerase II as an indicator of clinical response to adriamycin. *Anticancer Res.*, **11**, 429-432.

KUIPER CM, BROXTERMAN HJ, BAAS F, SCHUURHUIS GJ, HAISMA HJ, SCHEFFER GL, LANKELMA J AND PINEDO HM. (1990). Drug transport variants without P-glycoprotein overexpression from a human squamous lung cancer cell line after selection with doxorubicin. *J. Cell. Pharmacol.*, **1**, 35-41.

LEE M-S, WANG JC AND BERAN M. (1992). Two independent amsacrine-resistant human myeloid leukemia cell lines share an identical point mutation in the 170 kDa form of human topoisomerase II. *J. Mol. Biol.*, **223**, 837-843.

MARINI JC, MILLER KG AND ENGLUND PT. (1980). Decatenation of kinetoplast DNA by topoisomerase II. *J. Biol. Chem.*, **255**, 4976-4979.

MATSUO K-L, KOHNO K, TAKANO H, SATO S-I, KIEU A AND KUWANO M. (1990). Reduction of drug accumulation and topoisomerase II activity in acquired teniposide resistant human KB cell lines. *Cancer Res.*, **50**, 5819-5824.

MILLER SA, DYKES DD AND POLESKY HF. (1988). A simple salting out procedure for extracting DNA from human nucleated cells. *Nucleic Acids Res.*, **16**, 215.

NEGRI C, CHIESA R, CERINO A, BESTAGNO M, SALA C, ZINI N, MARALDI NM AND ASTALDI RICOTTI GCB. (1992). Monoclonal antibodies to human DNA topoisomerase I and the two isoforms of DNA topoisomerase II: 170 and 180 kDa isozymes. *Exp. Cell Res.*, **200**, 452-459.

NELSON EM, TEWEY KM AND LIU LF. (1984). Mechanism of antitumor drug action: poisoning of mammalian DNA topoisomerase II on DNA by 4'-(9-acridinyl-amino)-methanesulphon-m-aniside. *Proc. Natl Acad. Sci. USA*, **81**, 1361-1365.

NITISS JL, LIU Y-X AND HSIUNG Y. (1993). A temperature sensitive topoisomerase I allele confers temperature dependent drug resistance on amsacrine and etoposide: a genetic system for determining the targets of topoisomerase II inhibitors. *Cancer Res.*, **53**, 89-93.

PARCHMENT RE, SOLEIMANPOUR K, PETROSE S AND MURPHY MJ Jr. (1992). Pharmacologic validation of human tumor clonogenic assays based on pleiotropic drug resistance: implications for individualized chemotherapy and new drug screening programs. *Int. J. Cell Cloning*, **10**, 359-368.

PETROV P, DRAKE FH, LORANGER A, HUANG W AND HANCOCK R. (1993). Localization of DNA topoisomerase II in Chinese hamster fibroblasts by confocal and electron microscopy. *Exp. Cell Res.*, **204**, 73-81.

POMMIER Y, KERRIGAN D, SCHWARTZ RE, SWACK JA AND McCURDY A. (1986). Altered DNA topoisomerase II activity in Chinese hamster cells resistant to topoisomerase II inhibitors. *Cancer Res.*, **46**, 3075-3081.

PROSPERI E, SALA E, NEGRI C, OLIANI C, SUPINO R, ASTRALDI RICOTTI GBC AND BOTTIROLI G. (1992). Topoisomerase II α and β in human tumor cells grown *in vitro* and *in vivo*. *Anticancer Res.*, **12**, 2093-2100.

SAMBROOK J, FRITSCH EF AND MANIATIS T. (1989). *Molecular Cloning: a Laboratory Manual*, 2nd edn. Cold Spring Harbor Laboratory Press: Cold Spring Harbor, NY.

SCHNEIDER E, HORTON JK, YANG C-H, NAKAGAWA M AND COWAN KH. (1994). Multidrug resistance-associated protein gene overexpression and reduced drug sensitivity of topoisomerase II in a human breast carcinoma MCF7 cell line selected for etoposide resistance. *Cancer Res.*, **54**, 152-158.

SCHWARTZ DC AND CANTOR CR. (1984). Separation of yeast chromosome-sized DNAs by pulsed field gel electrophoresis. *Cell*, **37**, 67-75.

TAAGEPERA S, RAO PN, DRAKE FH AND GORBSKY GJ. (1993). DNA topoisomerase IIα is the major chromosome protein recognized by the mitotic phosphoprotein antibody MPM-2. *Proc. Natl Acad. Sci. USA*, **90**, 8407-8411.

TAKANO H, KOHNO K, ONO M, UCHIDA Y AND KUWANO M. (1991). Increased phosphorylation of DNA topoisomerase II in etoposide-resistant mutants of the human cancer KB cells. *Cancer Res.*, **51**, 3951-3957.

TAN KB, MATTERN MR, ENG W-K, McCABE FL AND JOHNSON RK. (1989). Nonproductive rearrangement of DNA topoisomerase I and II genes: correlation with resistance to topoisomerase inhibitors. *J. Natl Cancer Inst.*, **81**, 1732-1735.

TSAI-PFLUGFELDER M, LIU LF, LIU AA, TEWEY KM, WHANG-PENG J, KNUTSEN T, HUEBNER K, CROCE CM AND WANG JC. (1988). Cloning and sequencing of cDNA encoding human topoisomerase II and localization of the gene to chromosome region 17q21-22. *Proc. Natl Acad. Sci. USA*, **85**, 7177-7181.

UEDA K, CORNWELL MM, GOTTESMAN MM, PASTAN I, RONINSON IB, LING V AND RIORDAN JR. (1986). Expression of a full-length cDNA for the human '*mdr1*' gene confers resistance to colchicine, doxorubicin and vinblastine. *Biochem. Biophys. Res. Commun.*, **141**, 956-962.

UEMURA T, OKHURA H, ADACHI Y, MORINO K, SHIOZAKI K AND YANAGIDA M. (1987). Topoisomerase II is required for condensation and separation of mitotic chromosome in *S. pombe*. *Cell*, **50**, 917-925.

VAN DER SCHANS GP, VAN LOON AAWM, GROENENDIJK RH AND BAAN RA. (1989). Detection of DNA damage in cells exposed to ionizing radiation by use of anti-single-stranded DNA monoclonal antibody. *Int. J. Radiat. Biol.*, **55**, 747-760.

VAN DER SCHANS GP. (1993). Method for detecting single-stranded breaks in DNA. European patent request, No. 93201672.8, 10 June 1993.

VAN DER ZEE AG, DE JONG S, KEITH WN, HOLLEMA H, BOONSTRA H AND DE VRIES EGE. (1994). Quantitative and qualitative aspects of topoisomerase I and IIα and β in untreated and platinum/cyclophosphamide treated malignant ovarian tumors. *Cancer Res.*, **54**, 749-755.

VAN LOON AAWM, GROENENDIJK RH, TIMMERMAN AJ, VAN DER SCHANS GP, LOHMAN PHM AND BAAN RA. (1992). Quantitative detection of DNA damage after exposure to ionizing radiation by means of an improved immunochemical assay. *Mutat. Res.*, **274**, 19-27.

VERSANTVOORT, CHM, BROXTERMAN HJ, PINEDO HM, FELLER N, KUIPER CM AND LANKELMA J. (1992). Energy-dependent processes involved in reduced drug accumulation in multidrug-resistant human lung cancer cell lines without P-glycoprotein overexpression. *Cancer Res.*, **52**, 17-23.

VOLM M AND MATTERN J. (1992). Expression of topoisomerase II, catalase, methallothionine and thymidylate-synthase in human squamous lung carcinomas and their correlation with doxorubicin resistance and with patients' smoking habits. *Carcinogenesis*, **13**, 1947-1950.

WANG JC. (1985). DNA topoisomerases. *Annu. Rev. Biochem.*, **54**, 665-697.

WEBB CD, LATHAM, MD, LOCK RB AND SULLIVAN DM. (1991). Attenuated topoisomerase II content directly correlates with a low level of drug resistance in a Chinese hamster ovary cell line. *Cancer Res.*, **51**, 6543-6549.

WOESSNER RD, MATTERN MR, MIRABELLI CK, JOHNSON RK AND DRAKE FH. (1991). Proliferation and cell cycle-dependent differences in expression of the 170 kilodalton and the 180 kilodalton forms of DNA topoisomerase II in NIH-3T3 cells. *Cell Growth Different.*, **2**, 209-214.

ZAMAN GJR, FLENS MJ, VAN LEUSDEN MR, DE HAAS M, MÜLDER HS, LANKELMA J, PINEDO HM, SCHEPER RJ, BAAS F, BROXTERMAN HJ AND BORST P. (1994). The human multidrug resistance-associated protein (MRP) is a plasma membrane drug efflux pump. *Proc. Natl Acad. Sci. USA*, **91**, 8822-8826.

ZINI N, SANTI S, OGNIBENE A, BAVELLONI A, NERI LM, VALMORI A, MARIANI E, NEGRI C, ASTALDI-RICOTTI GCB AND MARALDI NM. (1994). Discrete localization of different DNA topoisomerases in HeLa and K562 cell nuclei and subnuclear fractions. *Exp. Cell Res.*, **210**, 336-348.